# Are Books Like Number Lines? Children Spontaneously Encode Spatial-Numeric Relationships in a Novel Spatial Estimation Task

**DOI:** 10.3389/fpsyg.2017.02242

**Published:** 2017-12-21

**Authors:** Clarissa A. Thompson, Bradley J. Morris, Pooja G. Sidney

**Affiliations:** ^1^Department of Psychological Sciences, Kent State University, Kent, OH, United States; ^2^Department of Educational Psychology, Kent State University, Kent, OH, United States

**Keywords:** spatial-numeric association, numerical representation, magnitude knowledge, number line estimation, numeracy, literacy

## Abstract

Do children spontaneously represent spatial-numeric features of a task, even when it does not include printed numbers (Mix et al., 2016)? Sixty first grade students completed a novel spatial estimation task by seeking and finding pages in a 100-page book without printed page numbers. Children were shown pages 1 through 6 and 100, and then were asked, “Can you find page X?” Children’s precision of estimates on the page finder task and a 0-100 number line estimation task was calculated with the Percent Absolute Error (PAE) formula (Siegler and Booth, 2004), in which lower PAE indicated more precise estimates. Children’s numerical knowledge was further assessed with: (1) numeral identification (e.g., What number is this: 57?), (2) magnitude comparison (e.g., Which is larger: 54 or 57?), and (3) counting on (e.g., Start counting from 84 and count up 5 more). Children’s accuracy on these tasks was correlated with their number line PAE. Children’s number line estimation PAE predicted their page finder PAE, even after controlling for age and accuracy on the other numerical tasks. Children’s estimates on the page finder and number line tasks appear to tap a general magnitude representation. However, the page finder task did not correlate with numeral identification and counting-on performance, likely because these tasks do not measure children’s magnitude knowledge. Our results suggest that the novel page finder task is a useful measure of children’s magnitude knowledge, and that books have similar spatial-numeric affordances as number lines and numeric board games.

## Introduction

Do children spontaneously represent spatial-numeric features of a task, even when it does not include printed numbers (Mix et al., 2016)? Previous research has provided evidence of spatial-numeric associations early in development suggesting that space and number share a common representational format (McCrink and Opfer, 2014; Patro et al., 2014). We investigated the possibility that books have spatial-numeric affordances like number lines and board games. Specifically, all three share left-to-right orientations, promote equal spacing between values, and provide an explicit means for mapping numbers to relative magnitudes (see **Table 1**). The overarching goal of the present experiment was to compare children’s performance on tasks known to tap numerical knowledge to a novel measure, the page finder task, which asked children to estimate the location of a page within a book without labeled page numbers and is hypothesized to measure magnitude estimation. If book affordances are related to the affordances of other measures, such as number lines, then the results of the page finder task should be highly related to other measures that tap children’s numerical magnitude understanding.

**Table 1:** Comparison of magnitude affordances across materials.

Affordance	Number line	Linear board game	Books
Orientation: Magnitudes increase from left-to-right	Left side is zero value and right side is maximum value (e.g., 100)	Starting space (on left side) is one and final space is maximum value (e.g., 10 or 100)	As pages are flipped, pages with smaller numbers are placed on the left and pages with larger numbers remain on the right
Linearity/Movement: Equal distance between moves/Individual moves represent the same distance	Each hatch mark represents equal value/moving from five to six on the number line is equivalent to moving from 55 to 56	Each space on game board represents one value/Each space is an equivalent move	Each page represents two values (front and back)/Each page turn is an equivalent move
Spatial/Temporal: Increasing physical space between locations (or time to reach location) indicates larger magnitudes	When starting from 0, finding and marking larger numbers (e.g., 73) takes longer than smaller numbers (e.g., 11), and there is a larger physical distance between 0 and the larger number (e.g., 11 units between 0 and 11 and 73 between 0 and 73).	Moving to spaces farther from the initial space takes longer amounts of time than moving to spaces closer to the initial space	From first page, finding a page with a larger number (e.g., 73) takes a longer time than finding a page with a smaller number (e.g., 11)

### Numbers and Space

Number sense refers to representing and processing numbers and includes several underlying processes, such as the ability to subitize a small number of items exactly, count, and compare approximate values (Dehaene, 2011; Friso-van den Bos et al., 2014). Children’s number sense becomes formalized as they map number words onto a mental number line via cultural tools (e.g., number lines; Thompson and Opfer, 2010). As children get older and gain more experience with numbers, they increasingly differentiate the underlying spatial-numeric representations into more precise number concepts (e.g., 75 is bigger than 35; Siegler, 2016), and this precision is predictive of concurrent and future performance on standardized mathematics achievement tests (Siegler et al., 2011; Starr et al., 2013; Fazio et al., 2014; Siegler and Thompson, 2014).

Numbers are represented both as approximate magnitudes and as exact categories such as “five” (Dehaene, 2011). Comparisons of approximate magnitudes are faster and more accurate as the ratio of difference between numbers increases (e.g., the numerical distance effect), and this provides evidence for spatial-numeric associations (Dehaene, 2011). According to the numerical distance effect, participants are faster and more accurate when deciding that 4 is larger than 1 than when deciding that 3 is larger than 2 because the mental representations for 4 and 1 overlap to a lesser degree than do the mental representations for 3 and 2. Thus, 4 and 1 are more distant and discriminable from one another than are 3 and 2.

Approximate number magnitudes are represented in a left-to-right ascending order along a mental number line in which small numbers are oriented on the left and large numbers are oriented on the right (Siegler and Opfer, 2003; Siegler, 2016). Evidence for spatial-numeric associations in children (van Galen and Reitsma, 2008), adults (Fias, 2001), and even chimpanzees (Adachi, 2014), comes from the investigation of the SNARC effect (Spatial Numerical Association of Response Codes) in which response rates are faster for relatively small numbers (0–4) when responses are made with the left hand and faster for large numbers (5–9) when responses are made with the right hand (Dehaene et al., 1993; Wood et al., 2008). The SNARC effect demonstrates a response bias consistent with a mental number line in which numbers increase in magnitude from left-to-right in cultures with left-to-right orthographies (Dehaene, 2011).

As further evidence of the spatial-numeric association in children, even young preschoolers show an advantage on numerical tasks that have an orientation that is consistent with the left-to-right directionality of writing in their cultures. For instance, United States children played a spatial search match-to-sample game in which they were shown two boxes with seven compartments each. The compartments in the sample and matching box were verbally labeled in an increasing numeric order from left-to-right or right-to-left. In the game, children were shown an object hidden in one of the compartments in the sample box, and they were asked to find another object that was hidden in the same numbered compartment in the matching box. Children were faster and more accurate at finding the hidden object in the matching box if both boxes were verbally numbered from left-to-right as compared to right-to-left (Opfer et al., 2010). Further, those children who spontaneously counted an array of ten chips from left-to-right, added one chip to the right side of a row of three chips, and took away one chip from the right side of a row of four chips were more likely to accurately give a researcher a specified number of chips in the typical Give-N task (e.g., Can you give me 8 chips?) as compared to those children who did not display this spatial-numeric association (Opfer et al., 2010).

### Spatial-Numeric Features of the Number Line and Cues that Co-vary with Number

Given the spatial-numeric nature of children’s numerical representations (i.e., the mental number line), the number line estimation task has emerged as a robust (e.g., Laski and Siegler, 2007; Booth and Siegler, 2008; Opfer and Thompson, 2008; Thompson and Opfer, 2008, 2010, 2016) and predictive (e.g., Booth and Siegler, 2006; Siegler et al., 2011, 2012; Siegler and Thompson, 2014) measure of children’s underlying numerical representations. In the number line estimation task, participants are shown a left endpoint labeled with 0 and a right endpoint labeled with a much larger number, such as 100. Participants’ job is to estimate the location of a third number on the line by making a vertical hatch mark. Initially, numerical representations, as measured by the number line estimation task, are characterized by even (i.e., linear) spacing across smaller numeric ranges and compression across larger numeric ranges (see Siegler et al., 2009 for a review). For instance, second graders make accurate, linear estimates in the 0–100 range and less precise estimates in the 0–1,000 range (Siegler and Opfer, 2003). These children are not only more accurate in their small-scale estimates, they are also more *confident* in their small-scale as opposed to large-scale estimates (Wall et al., 2016). As children gain experience or receive corrective feedback on their estimates, they show linear spacing across increasingly larger numeric ranges (Opfer and Siegler, 2007; Opfer and Thompson, 2008; Thompson and Opfer, 2008, 2016), however, even adults continue to struggle to produce linear estimates in some very large numeric ranges. That is, only about half of adults make accurate, linearly spaced estimates in the 0 – billion numeric range (Landy et al., 2013). It should be noted that there has been a recent debate about the shape of children’s numerical representations, and proponents of the proportion judgment account (e.g., Barth and Paladino, 2011; Slusser et al., 2013) suggest that a cyclical power function fits children’s estimates better than a logarithmic or a linear function. However, proponents of the logarithmic-to-linear shift account (Opfer et al., 2011, 2016) suggest that providing children with feedback about the number located at the midpoint of a 0–1,000 number line anchors their estimates to 500, thus making the fit of the cyclical power function to children’s number line estimates an artifact of the experimental methodology used. In the current paper, however, it is not our goal to make claims about children’s conceptual change in number line estimation tasks (e.g., best fitting function that characterizes children’s underlying numerical representation).

**FIGURE 1:**
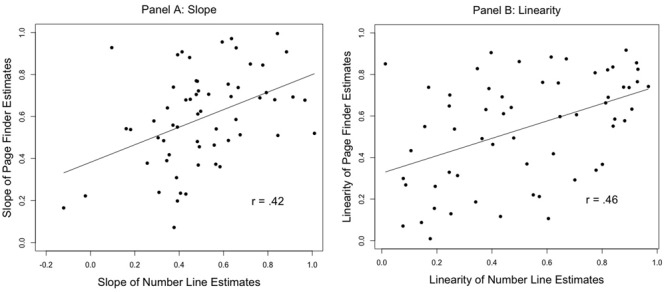
The slope of children’s number line estimates were significantly related to the slope of children’s page finder estimates **(A)**. Furthermore, the linearity of children’s number line estimates were significantly related to the linearity of children’s page finder estimates **(B)**.

The number line task has both spatial and numeric components. There are numerically labeled end-points on the number line as well as a to-be-estimated number that appears above the number line. To estimate the magnitudes appropriately, the child needs to map the to-be-estimated number to the correct spatial location (i.e., distance from the left and right end point). Sidney et al. (2017, see Figure 1 from their paper) suggest that, at a minimum, children must employ cross-format proportional reasoning to make accurate, linear estimates of where given numbers are located on number lines, for example, in a typical number-to-position task (Siegler and Opfer, 2003). In this task, children are shown a line segment with symbolic anchors of 0 and 100 at the endpoints, and children’s job is to find where along the line the to-be-estimated number is located. For instance, to accurately place 78 on a 0–100 number line, a child must estimate the length of a line segment that is 78% of the distance of the 100-unit line. To do so, a child must consider the ratio of the numerical magnitudes of 78 and 100 and match that ratio to the spatial magnitude of the 0–100 number line to estimate the spatial magnitude of a 0–78 line segment (see Barth and Paladino, 2011). The number line estimation task is a prime example of how space and number are naturally integrated. To accurately complete the number line estimation task, participants must map an internal numerical magnitude representation to an external physical location on the line. Children who have a more precise mapping between their internal numerical magnitude representation and external spatial extent make more accurate number line estimates.

### Improving Number Sense

Improving children’s estimates on the number line task appears to improve the precision of children’s mental number line, because improvements transfer to other types of tasks. In interventions aimed at improving children’s number line estimates (Opfer and Siegler, 2007; Opfer and Thompson, 2008; Thompson and Opfer, 2008, 2016), children were provided with corrective feedback about the location of the number 150 on a 0–1,000 number line. The feedback alerted children to the fact that their estimates were quite far from the correct location of 150 on the number line. Subsequently, the children scaled their estimates across the entire 0–1,000 numeric range based on their new knowledge of the correct location for 150. To investigate the robustness of this newly adopted linear representation, children were presented with a magnitude categorization transfer task (Opfer and Thompson, 2008). In the magnitude categorization task, five boxes were arranged from left-to-right with a box labeled “really small” for numbers like 0 on the far left and a box labeled “really big” for numbers like 1,000 on the far right. Interestingly, children who made a linear series of number line estimates also made a linear series of category judgments, and this suggests that the linear representation had transferred from one numerical context to another. The left-to-right orientation of the number line and categorization task was aligned with the left-to-right orientation of children’s mental number line.

In addition to intervening more directly by providing one-on-one feedback on children’s estimates on the number line task, a variety of interventions have aimed to improve children’s number sense in more ecologically natural contexts (e.g., board games). There has been increasing interest in improving mathematics performance in early school years by improving children’s number sense, through formal and informal instruction (Berkowitz et al., 2015; Ramani and Siegler, 2015; Fazio et al., 2016; Hamdan and Gunderson, 2017). These interventions suggest that learning is improved when the affordances of materials are aligned with the properties of the mental number line (Siegler, 2016). Next, we review recent research on the use of board games to improve children’s mathematics performance.

Interventions using board games have demonstrated learning benefits for children (Ramani and Siegler, 2008; Siegler and Ramani, 2008, 2009; Whyte and Bull, 2008; Ramani et al., 2012; Laski and Siegler, 2014). Board games provide overlapping cues for children to learn the relations between number words and their relative magnitudes (e.g., moving ten spaces from left-to-right takes the child more time to execute and a larger number of moves than moving two spaces from left-to-right). Children who played a board game with ten numbered spaces oriented from left-to-right made larger learning gains than children who played an analogous color board game without consecutively numbered spaces. Specifically, playing the board game for four 15 min sessions, in which smaller numbers were presented in spaces on the left and larger numbers were presented in spaces on the right, improved children’s numeral identification, number line estimation, and magnitude comparison performance (Ramani and Siegler, 2008; Siegler and Ramani, 2008). A subsequent experiment investigated the role of linearity in supporting learning by comparing the effects of a linear board game (i.e., spaces numbered 1–10 in a left-to-right orientation) and a circular board game (i.e., spaces numbered 1–10 in a clockwise orientation) (Siegler and Ramani, 2009). The results demonstrated larger learning gains for children who played the linear game because it was hypothesized that the linear board game was better aligned with children’s mental number line as compared to the circular board game.

Evidence from these board game experiments suggests that three affordances appear to be most important for materials that support learning number magnitudes: (1) left-to-right orientations, (2) promoting equal spacing between values, and (3) providing an explicit means for mapping numbers to relative magnitudes (Siegler and Booth, 2004; Whyte and Bull, 2008; Laski and Siegler, 2014; Ramani and Siegler, 2015).

### Spatial-Numeric Affordances of Books

Reading books to children is an important aspect of promoting children’s developing literacy. Sharing reading with young children promotes an understanding of reading conventions (e.g., orthography oriented from left-to-right and top-to-bottom; Whitehurst and Lonigan, 1998) and introduces children to skills related to later reading (e.g., phonemic awareness; Justice et al., 2005). Discussions during shared reading that prompt children to make inferences beyond text improve children’s vocabulary and comprehension (Zucker et al., 2013). Books not only support children’s developing literacy, but support their developing numeracy. Books can provide support for number and math learning by providing content (e.g., novel words) and opportunities for social interactions (Montag et al., 2015), learning number words (Ward et al., 2017), providing practice for number skills (Skwarchuk et al., 2014), learning relational quantity words like “equal, more, or less” (Hassinger-Das et al., 2015), and improving spatial reasoning (Gunderson et al., 2012). One heretofore unexamined dimension is that the affordances of the book may provide supports for spatial-numeric learning for relative magnitudes, much like number lines and board games.

The affordances of books may be analogous to number lines and linear board games because they provide overlapping cues for mapping number words to approximate magnitudes (see **Table 1** for comparisons). Recall that the left-to-right orientation of number lines and linear board games was related to greater increases in learning. Books are oriented left-to-right in a similar fashion with smaller page numbers on the left and increasingly larger page numbers on the right. Number lines promote equal spacing between values because the distance between end markers can be evenly divided by equally spaced hatch marks (see Siegler and Opfer, 2003; Schneider et al., 2008; Siegler et al., 2011; and Ashcraft and Moore, 2012, for children’s spontaneous segmentation of number lines and Siegler and Thompson, 2014; Peeters et al., 2017a,b for children’s use of experimenter-imposed landmarks as they estimated numbers on number lines). Linear board games are structured such that each space represents one value, and moves between spaces are all the same distance. Books have similar affordances in that each page contains two numbers, one on the front and one on the back of each page, and each page turn moves the same distance between the first and last page. Finally, number lines and linear board games provide a means for helping children map numbers to relative magnitudes.

### Current Study

In our current study, we created a novel page finder magnitude estimation task in which we asked children to find pages in a 100-page book that did not include printed page numbers. We anticipated that number line estimation performance in the 0–100 range would be related to performance on this page finder task because we oriented children to the book by verbally labeling the first six pages. For this reason, we expected that children might draw comparisons between the 23 cm wide number lines and the 1 cm wide book to decide that the book was simply a smaller, scaled-down version of the number line that did not include printed numeric labels. The classic literature on scale errors suggests that it is not uncommon for preschoolers to attempt to interact with small-scale objects (e.g., tiny replica of a car) in much the same way that they previously interacted with large-scale objects (e.g., large car) (DeLoache et al., 2004). Further, in the domain of mathematics, even infants and young children who do not have formal multiplication and division experience, can perform multiplicative scaling in a non-symbolic context (McCrink and Wynn, 2007; McCrink and Spelke, 2010, 2016). Finally, children transfer their knowledge of linearly arranged numbers to other non-numeric stimuli, such as their estimates of the locations of letters of the alphabet on an ABC line (Hurst et al., 2014).

Our sixty first grade participants completed five tasks in a counterbalanced order: number line estimation (e.g., Where does 25 go on a line with left endpoint labeled 0 and right endpoint labeled 100?), magnitude comparison (e.g., Which is bigger 89 or 54?), numeral identification (e.g., What number is this: 17?), counting on (e.g., Can you count up five more from 84?), and a page finder magnitude estimation task (e.g., Can you find page 33?). We hypothesized that: (1) Magnitude comparison, numeral identification, and counting on performance would be correlated with number line performance because all of these tasks tap numerical knowledge, and (2) To the extent that the page finder magnitude estimation task also taps magnitude understanding, number line estimation performance will predict page finder performance, even after controlling for age and accuracy on the magnitude comparison, numeral identification, and counting on tasks.

## Materials and Methods

### Participants

Participants were 60 first grade students (*M* age = 6.68, *SD* = 0.89) in four classrooms in two public school districts in northeast Ohio. Approximately 39% of children who attended these schools were eligible for free or reduced price lunches. Gender was balanced in the sample: 50% of children were identified as female. Parents provided written informed consent for their children to participate, and children provided verbal assent. Each child received a sticker at the end of the experimental session. The Kent State University IRB approved this study.

### Tasks and Procedure

Participants completed five tasks: number line estimation, numeral identification, counting on, magnitude comparison, and page finder magnitude estimation. The number line estimation task is a measure that assesses children’s magnitude knowledge; numeral identification is a task that measures children’s ability to verbally identify numbers in the 0–100 range that were presented in the other numerical tasks such as number line estimation and the novel page finder task; counting on is a measure that assesses children’s numerical knowledge such as the ability to make decade changes as they count; magnitude comparison is a measurement that assesses children’s ability to compare numbers in the 0–100 range, and we believed this would be important as children compared the current and previous pages that they found in the page finder task (e.g., “I just found page ___, and now I have to find a bigger page number, page ___.”) Task order was counterbalanced, and the problems were presented in a random order within each task. All children were tested individually in a quiet location in their school by a female research assistant.

#### Number Line Estimation

Children estimated the location of 24 numbers on 23 cm number lines. The lines had a 0 at the left endpoint and a 100 at the right endpoint. One to-be-estimated number appeared at the top left of each page. Children indicated the location of this number by making a vertical hatch mark through the line. When children finished making each estimate, the page was turned over so that they could no longer reference their answer. All children were first asked to point to the location of 0 and 100 and were provided with corrective feedback if they did not point to the correct locations. Then, they estimated the following numbers, that spanned the entire 0–100 range, without feedback from the researcher: 3, 4, 6, 8, 12, 17, 21, 23, 25, 29, 33, 39, 43, 48, 52, 57, 61, 64, 72, 79, 81, 84, 90, and 96. This set of numbers over-samples children’s estimates at the low end of the numerical range, consistent with prior research (Opfer et al., 2016). In line with prior research (e.g., Siegler and Booth, 2004; Laski and Siegler, 2007; Booth and Siegler, 2008; Laski and Siegler, 2014), we assessed three aspects of children’s estimates: their PAE, the linearity of children’s estimates, and the slope of their best fitting linear function. PAE is the absolute difference between the child’s estimate and the actual location of the number divided by the scale and expressed as a percentage (i.e., multiplied by 100). Smaller PAE indicates a more accurate series of estimates. Linearity and slope are calculated by regressing each child’s set of estimates on the true magnitude of the given numbers. The *R^2^*_Lin_ represents the percent of variance in each child’s estimates accounted for by the best fitting linear model for that child. The slopes (*b_j_*) of the best fitting linear model for each child indexes how close that child’s estimates are to the ideal slope that relates estimates to the given numbers (1.00). It should be noted that we chose to characterize children’s estimates with a linear function to maintain consistency with prior research on informal tasks (i.e., board games, Siegler and Ramani, 2008) associated with children’s number line estimates, however, there are other statistical methods for characterizing children’s behavior on this task (e.g., Barth and Paladino, 2011; see Opfer et al., 2016 for a discussion).

#### Numeral Identification

Children named 24 numbers, one at a time, as they were presented on a computer screen. The numbers were the same as those from the number line estimation task. The dependent variable was percentage correct out of 24 trials.

#### Counting On

This game was adapted from Laski and Siegler (2014) because “counting on” has been established as an important aspect of the typical numerical board game procedure (e.g., when children are on space 5, and they spin a 2, they must say, “6, 7” instead of “1, 2”). Children heard a number (7, 18, 37, and 84), and they were asked to count up by 3, 5, and 8 from each of those starting numbers (e.g., “7, 8, 9, 10, 11, 12”). They were first given the sample problem, “If I say, ‘Start counting with one and count up two more numbers,’ you would say, ‘1, 2, 3’.” To ease the working memory burden of the task, children were presented with a linear array of counting chips that corresponded to the number that they had to count up. They were shown the strategy of pointing to each chip as they counted, and they were reminded that they should say the first number and then point to each chip once as they said the next number in the sequence. The dependent variable was percentage correct out of 12 trials. The child could not make any counting errors on a trial for it to be counted as correct.

#### Magnitude Comparison

Participants were told that they would see two numbers between 0 and 100, and they should compare the numbers to decide which one was bigger. All comparisons contained the number 54, which was chosen because it is close to the midpoint of the 0–100 range (see Siegler et al., 2011 for a similar methodology used in a fraction magnitude comparison task). It was assumed that if children were asked to compare all numbers to 50, this would make the task too easy and would also provide unintended clues about the midpoint of the 0–100 numerical range. Then, children could potentially use these clues as feedback to improve their number line estimation performance (see Opfer et al., 2016). The following numbers were compared with 54: 2, 8, 12, 26, 34, 42, 67, 73, 89, 97. In half of the trials, 54 appeared on the left side of the screen, and in the other half of trials 54 appeared on the right side of the screen. The dependent variable was percentage correct out of 20 trials.

#### Page Finder Magnitude Estimation

Children were presented with a 100-page book. The book did not include any page numbers. The front and back cover of the book was white, and the book was spiral bound. The children were told that they were going to play a search game. The researcher said a number, and the child was instructed to flip to that page without counting. The researcher said, “Just like one of your books at home, Page 1 is on this side (researcher pointed to page), and Page 2 is on this side (researcher flipped the page and pointed to it). If Page 3 is on this side (researcher pointed to it), which page is on this side (researcher flipped the page and pointed to it)? When children answered correctly, they were told, “Good!” When children answered incorrectly, they were told, “It would be Page 4, right?”

The researcher continued with the instructions, “If this is Page 5 (researcher pointed to page) which page is on this side (researcher flipped the page and pointed to it)?” Again, children were given corrective feedback on this practice trial (i.e., “Good,” or “It would be Page 6, right?”). Then, the child was told, “The book keeps going until we get to page 100 (researcher flipped to page 100).” Then, the child was asked to find page 1 and page 100, and they were given corrective feedback if they did not correctly identify these practice pages.

Children did not receive any corrective feedback on the remaining test trials. They were told, “If I say the number ‘20,’ I want you to quickly flip the pages until you believe that you’ve gotten to page 20. See you can quickly flip through the pages like this.” The researcher demonstrated how to quickly fan through the pages. Children were reminded how to properly flip through the book if they attempted to count the pages. This most frequently happened when they were asked to find a small number page. It should be noted that some children chose to flip from the back of the book or lift a chunk of pages when the book was closed to get closer to the intended location of a large-numbered page in the book. According to our research assistant, flipping from the back of the book was rare, though admissible in our protocol.^1^ After the child flipped to the intended page, he was asked to find a hidden picture on the page. The researcher closed the book before the child searched for the location of the next page. Some children used the strategy of lifting a large chunk of pages to get to the back of the book if asked to find a large number page. We did not systematically code children’s flipping strategies for later analysis.

If children forgot the number of the page that they were looking for, the researcher could verbally remind them by saying, “Where is page N?” Children were asked to search for the following pages: 4, 8, 17, 23, 29, 33, 48, 57, 61, 72, 84, and 90. In line with the number line estimation task, we calculated percent absolute error, PAE = (|page number that the child flipped to – the actual page number|/ 100) ^∗^100, linearity (*R^2^*_Lin_), and slopes of the best fitting linear models.

## Results

First, we examined children’s average performance on each task, see **Table 2**. As shown in **Table 2**, children had high accuracy on the numeral identification, magnitude comparison, and counting on tasks, indicating knowledge of numerical symbols. Furthermore, on average, children’s number line estimates were moderately good, with an average PAE of 14%. However, there was substantial variability in the accuracy and precision of children’s number line estimates.

**Table 2:** Average performance in current and prior studies.

Task	Mean: current study	Mean: prior studies	Prior study citation
Number line estimation: PAE	18% (7%)	18% (1st) and 14% (1st); 13% (1st)	Siegler and Booth, 2004; Laski and Siegler, 2007
Number line estimation: *R^2^*_Lin_	0.52 (0.28)	0.49 (K), 0.90 (1st)	Siegler and Booth, 2004
Number line estimation: Slope	0.51 (0.23)	0.33 (K), 0.58 (1st)	Siegler and Booth, 2004
Numeral identification accuracy	96% (6%)	82% (K)	Laski and Siegler, 2014
Counting on accuracy	79% (27%)	18% (K)	Laski and Siegler, 2014
Magnitude comparison accuracy	94% (10%)	95% (1st)	Laski and Siegler, 2007
Page finder magnitude estimation: PAE	16% (5%)	N/A	N/A
Page finder magnitude estimation: *R^2^*_Lin_	0.54 (0.26)	N/A	N/A
Page finder magnitude estimation: Slope	0.59 (0.23)	N/A	N/A

*Standard deviations reported in parentheses along with corresponding means. Sample characteristics from prior research are reported in parentheses along with corresponding means; K, kindergarten; 1st, first grade.*

Importantly, children’s performance on most of these tasks was in line with findings from prior research using these tasks with similar age groups. As shown in **Table 2**, children’s accuracy on numeral identification and magnitude comparison was consistent with prior research with first graders and kindergartners (Laski and Siegler, 2007; Laski and Siegler, 2014). In light of the replicability crisis in psychology (Open Science Collaboration, 2015), we wanted to show that our results were consistent with the existing numerical cognition literature. Note that data were collected from our first grade participants in the early part of the academic year (i.e., October and November), and it is for this reason that their performance on some tasks may resemble that of kindergartners from the previous literature. Furthermore, children’s average error (PAE), linearity (R^2^_Lin_), and slopes on the number line task were also consistent with prior research (Siegler and Booth, 2004; Laski and Siegler, 2007, 2014). In contrast to prior literature, the children in our sample were more accurate on the counting on task compared to prior research demonstrating poor counting on performance among kindergartners (Laski and Siegler, 2014). Knowledge of the number system develops rapidly across kindergarten and first grade, and thus this difference in performance may reflect differences in the timing of data collection across the current study and prior research.

Second, we tested for correlations between accuracy on all pairs of tasks. Consistent with prior literature (Laski and Siegler, 2007; Ramani and Siegler, 2008; Siegler and Ramani, 2008, 2009), we expected that children’s accuracy on the numeral identification, counting on, and magnitude comparison tasks should be significantly correlated with children’s PAE on the number line estimation task. Indeed, this was the case, see **Table 3**. Across all three numeric tasks, lower PAE on the number line estimation task was associated with higher accuracy on the numeric tasks. In other words, as expected, children with more precise representations of whole number magnitude were also more likely to be adept at identifying printed numerals, counting up from a given number, and choosing the larger of two given numbers. Importantly, children’s PAE on the number line estimation task was also significantly correlated with PAE on our novel, page finder magnitude estimation task, *r* = 0.39, *p* < 0.01. Children’s PAE on the page finder task was also correlated with magnitude comparison, *r* = -0.27, *p* = 0.04, but not significantly correlated with the other numerical tasks that do not measure magnitude knowledge.

**Table 3:** Pairwise correlations between tasks.

Task	Numeral	Counting	Magnitude	Page
	identification	on	comparison	finder PAE
Number line estimation PAE	–0.49^∗∗^	–0.38^∗∗^	–0.57^∗∗^	0.39^∗∗^
Numeral identification accuracy		0.30^∗^	0.68^∗∗^	–0.18
Counting on accuracy			0.58^∗∗^	–0.07
Magnitude comparison accuracy				–0.27^∗^

*Single asterisks denote correlations significant at *p* < 0.05. Double asterisks denote correlations significant at *p* < 0.01. For correlations with Magnitude Comparison, *n* = 59, otherwise, *n* = 60.*

Given that the precision of children’s magnitude estimates during the number line task was highly correlated with the precision of children’s magnitude estimates during the page finder estimation task, we assessed whether the *linearity* (*R^2^*_Lin_) of their magnitude estimates and the *slope* of the best fitting lines were also similar across tasks. Both *R^2^*_Lin_, *r* = 0.46, *p* < 0.001, and slope, *r* = 0.42, *p* < 0.001, were correlated across tasks (see **Figure 1**). This is further evidence that children who made highly linear estimates on the number line were also likely to make highly linear estimates when seeking page numbers in a book.

Finally, we examined whether children’s magnitude estimation performance on the page finder task was related to their magnitude estimation performance on the number line estimation task, over and above the other facets of children’s numerical knowledge. Although all of the tasks assess children’s number knowledge, we hypothesized that the number line estimation task and the page finder magnitude estimation task would both specifically assess magnitude understanding, and therefore would be significantly related even after accounting for other aspects of children’s number knowledge. Thus, we regressed children’s PAE on the page finder task on children’s PAE on the number line task, controlling for accuracy on numeral identification, counting on, and magnitude comparison as well as age. In this model, children’s number line estimation PAE did predict their page finder PAE, β = 0.48, *p* < 0.01, $\eta_{p}^{2}$ = 0.14. In contrast, numeral identification, *p* = 0.41, $\eta_{p}^{2}$ = 0.01, counting on, *p* = 0.20, $\eta_{p}^{2}$ = 0.03, and magnitude comparison, *p* = 0.21, $\eta_{p}^{2}$ = 0.03, did not predict children’s PAE on the page finder task in this model.

## Discussion

Our results provided evidence for a novel measure of spatial-numerical association, the page finder task. We found that for sixty first grade students, their performance on a number line estimation task in the 0–100 range was correlated with their performance on other numerical tasks, such as magnitude comparison, numeral identification, and counting on from a given number. Importantly, all three dependent variables that characterized performance in the number line estimation task (i.e., PAE, *R^2^*_Lin_, and slope) were related to the same dependent variables in the novel, page finder magnitude estimation task in which children were asked to find the location of a page number in a book. Interestingly, page finder PAE did not correlate with children’s accuracy on identifying numerals and counting on from a given number—tasks that seem to rely less on *magnitude* knowledge and more on *symbolic* numerical knowledge—and this may be related to the non-symbolic nature of the page finder book because it contained no printed page numbers. Children’s performance on the number line estimation task predicted their page finder PAE, even after controlling for overall age and performance on all other tasks tapping numerical knowledge. Overall, these findings suggest that children may be relying on similar mental representations to guide their estimates on both the highly symbolic number line estimation task and our novel page finder magnitude estimation task that contained no printed numbers.

It was somewhat surprising that children were just as accurate (i.e., similar PAEs and SDs) at finding page numbers in a book without printed page numbers as placing numbers on number lines. The number line estimation task can test for spatial-numeric associations because this task inherently involves spatial (e.g., identifying the physical location of a number on a number line as a distance between the left and right endpoints) as well as numeric components (e.g., end points on the number line, to-be-estimated numbers). Children’s accuracy on the page finder task was all the more impressive because the number line was 23 cm wide, yet the book used in the page finder task was only about 1 cm wide. We interpret children’s similar level of accuracy on these tasks as indicating that the number line estimation task and the page finder task tap a common underlying numerical representation. In this way, PAE on each task might indicate the level of precision in the underlying numerical representation: if participants’ numerical representations are precise enough to be accurate on one task, they are equally precise and accurate on the other. Was children’s performance so accurate on the page finder task because we oriented them to the size of one unit–a procedure similar to that used when children make estimates on “unbounded” number line tasks (Cohen and Sarnecka, 2014)–by orienting them to the first six pages in the book to make sure they understood the task instructions? Similarly, in the zips task (Booth and Siegler, 2006; Thompson and Siegler, 2010), children were shown the length of a 1-unit line and the length of a 1,000-unit line and asked to produce a line of X units. Performance on the zips task correlates with performance on the number line estimation task and a numerosity estimation task in which children fill a jar with a specified number of dots. Performance on these production tasks, such as the page finder, zips, and jar tasks, may all tap children’s underlying numerical magnitude representations, much like the number line estimation task.

In our regression analysis, we were able to predict page finder PAE from number line estimation PAE after controlling for age and performance on other numerical tasks, and we take this as evidence in support of the hypothesis that the page finder task and the number line estimation task tap a common underlying numerical representation. It is important to note that we are not able to make any causal claim about the direction of this relationship. In this analysis, we operationalized children’s underlying numerical representation by measuring their percent absolute error on the number line estimation task. Thus, we argue that our findings demonstrate that finding a page number in a book taps children’s underlying magnitude representation. If this is the case, it may be possible that finding page numbers in books is one way in which parents can help children improve their understanding of relative numerical magnitudes. Parents and teachers already encourage children’s literacy development through reading, and reading books is a familiar activity for many children. Our findings suggest that while reading books, caregivers can help children identify page numbers in the books in an effort to promote their understanding of numerical magnitudes. Like board game interventions, books may have the potential to provide an easy and cost-effective means for caregivers to integrate numerical experience into children’s everyday lives. In this way, books can promote the development of literacy as well as numeracy skills.

## Conclusion

Number sense is inherently spatial and numeric (Mix et al., 2016; Leibovich et al., 2017). We investigated whether books share similar spatial-numeric properties of materials, such as number lines, by using a novel measure, the page finder task. Our results demonstrated strong similarities between page finder estimates and children’s number line estimates, which is particularly impressive given that the page finder book was quite small (approximately 1 cm wide) in comparison to the number line. The findings demonstrate the utility of this novel measure and suggest that books share properties with other materials that measure, and potentially improve, children’s numerical magnitude knowledge.

## Ethics Statement

This study was carried out in accordance with the recommendations of the Kent State University IRB with written informed consent from all subjects. All subjects gave written informed consent in accordance with the Declaration of Helsinki. The protocol was approved by the Kent State University IRB.

## Author Contributions

CT and BM conceptualized the study. CT and BM oversaw the data collection with undergraduate research assistants. CT and PS analyzed the data. CT, BM, and PS wrote and revised the paper.

## Conflict of Interest Statement

The authors declare that the research was conducted in the absence of any commercial or financial relationships that could be construed as a potential conflict of interest.

## Footnotes

1 We would like to thank a reviewer for suggesting that flipping from the back of the book is parallel to estimating from the right (large) endpoint on the number line estimation task.
